# Cardiomyocytes Derived from Human ^Cardiopoietic^Amniotic Fluids

**DOI:** 10.1038/s41598-018-30537-z

**Published:** 2018-08-13

**Authors:** Angela Di Baldassarre, Maria A D’Amico, Pascal Izzicupo, Giulia Gaggi, Simone Guarnieri, Maria A Mariggiò, Ivana Antonucci, Barbara Corneo, Dario Sirabella, Liborio Stuppia, Barbara Ghinassi

**Affiliations:** 1 Department of Medicine and Aging Sciences, University “G. d’Annunzio” of Chieti-Pescara, Via dei Vestini 31, 66100 Chieti, Italy; 2 Department of Neuroscience, Imaging e Clinical Scienes, University “G. d’Annunzio” of Chieti-Pescara, Via dei Vestini 31, 66100 Chieti, Italy; 3 Department of Department of Psychological, Humanities and Territorial Sciences, University “G. d’Annunzio” of Chieti-Pescara, Via dei Vestini 31, 66100 Chieti, Italy; 4 Stem Cell Core Facility, Columbia University Medical Center, 650 W. 168th St., 10032 New York, NY USA

## Abstract

Human amniotic fluid (hAF) cells share characteristics of both embryonic and adult stem cells. They proliferate rapidly and can differentiate into cells of all embryonic germ layers but do not form teratomas. Embryoid-bodies obtained from hAF have cardiac differentiation potential, but terminal differentiation to cardiomyocytes (CMs) has not yet been described. Our purpose was to promote cardiac differentiation in hAFcells. Cells were exposed to inducing factors for up to 15 days. Only the subset of hAF cells expressing the multipotency markers SSEA4, OCT4 and CD90 (^Cardiopoietic^AF cells) responded to the differentiation process by increasing the expression of the cardiac transcription factors Nkx2.5 and GATA4, sarcomeric proteins (cTnT, α-MHC, α-SA), Connexin43 and atrial and ventricular markers. Furthermore, differentiated cells were positive for the calcium pumps CACNA1C and SERCA2a, with approximately 30% of ^Cardiopoietic^AF-derived CM-like cells responding to caffeine or adrenergic stimulation. Some spontaneous rare beating foci were also observed. In conclusion, we demonstrated that ^Cardiopoietic^AF cells might differentiate toward the cardiac lineage giving rise to CM-like cells characterized by several cardiac-specific molecular, structural, and functional properties.

## Introduction

Cardiovascular (CV) diseases are the main cause of mortality in the industrialized world, with an estimated 17.7 million deaths by CV in 2015, representing 31% of all global deaths^1^. Therefore, studies on CV biology, disease modeling, drug discovery and regenerative medicine represent a priority and an unmet medical need^2,3^. The prospect of repairing an injured heart with cells that can be cultured and expanded *ex vivo* and then functionally integrated upon transplantation is appealing. Moreover, the availability of *in vitro* human models of cardiac disorders reflecting human disease phenotypes has become crucial for the discovery and development of therapeutics. Indeed, much of our knowledge on the molecular pathways leading to human CV disorders has been derived from animal models^4,5^, but considerable differences exist between human and mouse genomes, and species-specific physiological properties lead to considerable functional differences^6,7^. To generate stem cell models of human CV disease and foster advances in regenerative medicine, it is critical to be able to generate and expand human CV progenitors or terminally differentiated, functional cardiac cells. Different types of stem cells have already been shown to have cardiomyogenic potential^8,9^: For example, embryonic stem (ES) cells and induced pluripotent stem (iPS) cells can be differentiated into beating cells with a cardiac-like phenotype *in vitro*. iPS cells are patient specific and may be used to avoid the immunological and ethical concerns of ES cells^10,11^, but their clinical use may be tempered by their tumorigenic potential^12^. Despite these encouraging premises, a safe source of cardiomyocytes (CMs) or CM progenitor cells to use for cell replacement therapy of damaged cardiac muscle tissue is still not available.

Mid-trimester human amniotic fluid (hAF) cells are a heterogeneous population that includes stem cells with intermediate features between embryonic and adult stem cells: indeed, they are characterized by a high proliferation rate, the expression of both pluripotent and mesenchymal markers and the ability to differentiate into lineages representative of all three germ layers, while maintaining the non-tumor-forming properties of adult cells^13^. Previous studies have already demonstrated that hAF cells express several cardiac-associated genes and that these cells can be induced along the cardiac lineage by modulating WNT signaling or by exposure to demethylating agents^14–16^. Nonetheless, it is well established that this differentiation process occurs with a very low yield. Hence, enabling high-throughput cardiogenesis in hAF cells has obvious therapeutic potential. However, hAF contains diverse cellular subpopulations. The percentage of cells expressing pluripotency markers varies greatly among reports from different labs^17,18^. For example, while several groups used CD117 (c-Kit, type III tyrosine kinase receptor for stem cell factor) to isolate the undifferentiated population^19,20^, other authors failed to select CD117^+^ cells from hAFs^20,21^; in addition, there is evidence that unselected AF and CD117^+^-isolated cells differ in both differentiation potential and efficacy^14,22^.

The aim of this study was to dissect and promote the cardiomyogenic potential of hAF cells to obtain differentiated progenies with morphological and functional features of CM. For this purpose, 15 hAF samples were analyzed for the expression of embryonic markers and then induced to cardiac differentiation through two differentiation protocols: one based on Embryoid Body (EB) formation and the other in which cell monolayers were sequentially exposed to known cardiac differentiation inducers.

## Results

### Phenotypical characterization of hAF samples

hAFs, isolated as previously described^16^, were characterized by flow cytometry between passages 6 and 8. As in previous studies^15,18,20^, we demonstrated that they expressed the mesenchymal stem cell markers CD29, CD73 and CD44, whereas they were negative for the hematopoietic markers CD34 and CD45 and for the endothelial marker PECAM-1/CD31. On the other hand, positivity for the expression of the pluripotent stem cell markers SSEA4, OCT4, Tra-1–60 and CD90 was extremely variable among samples (Table 1 and Supplemental Table 1).

**Table 1:** Characterization of AF cells samples.

AF Sample	Age at pregnancy (yr)	Time of Amniocentesis (Weeks of Gestatiion)	Fetal Karyotype	AF cell phenotyping (%)			EBs formation
				SSEA4	OCT4	CD90	
**#1**	**38**	**16**	**46, XY**	**65.3**	**60.2**	**84.1**	**+**
#2	38	16	46, XY	9.8	13.5	50.1	−
#3	37	16	46, XY	9.9	3.8	9.1	−
**#4**	**38**	**16**	**46, XY**	**54.5**	**15.4**	**93.5**	**+**
**#5**	**40**	**16**	**46, XY**	**82.2**	**73.7**	**48.1**	**+**
#6	40	17	46, XY	n.d.	9.9	n.d.	−
#7	42	16	46, XY	5.7	6.7	43.3	−
#8	41	16	46, XY	6.3	2.4	10.2	−
**#9**	**39**	**16**	**46, XY**	**80.4**	**12.5**	**64.6**	**+**
**#10**	**35**	**17**	**46, XY**	**59.1**	**78.5**	**45.6**	**+**
#11	41	16	46, XY	25.1	n.d.	7.8	−
#12	38	16	46, XY	9.9	6.8	51.3	−
#13	38	16	46, XY	6.8	7.4	5.7	−
**#14**	**32**	**17**	**46, XY**	**63.9**	**12.1**	**90.0**	**+**
**#15**	**35**	**17**	**46, XY**	**56.8**	**82.7**	**81.1**	**+**

In bold are highlighted the AF cells samples (^Cardiopioetic^AF cells) able to give rise to EB-like aggregates.

n.d.: not detectable.

### Samples with a pluripotency profile give rise to EBs with cardiac differentiation potential

**Figure 1:**
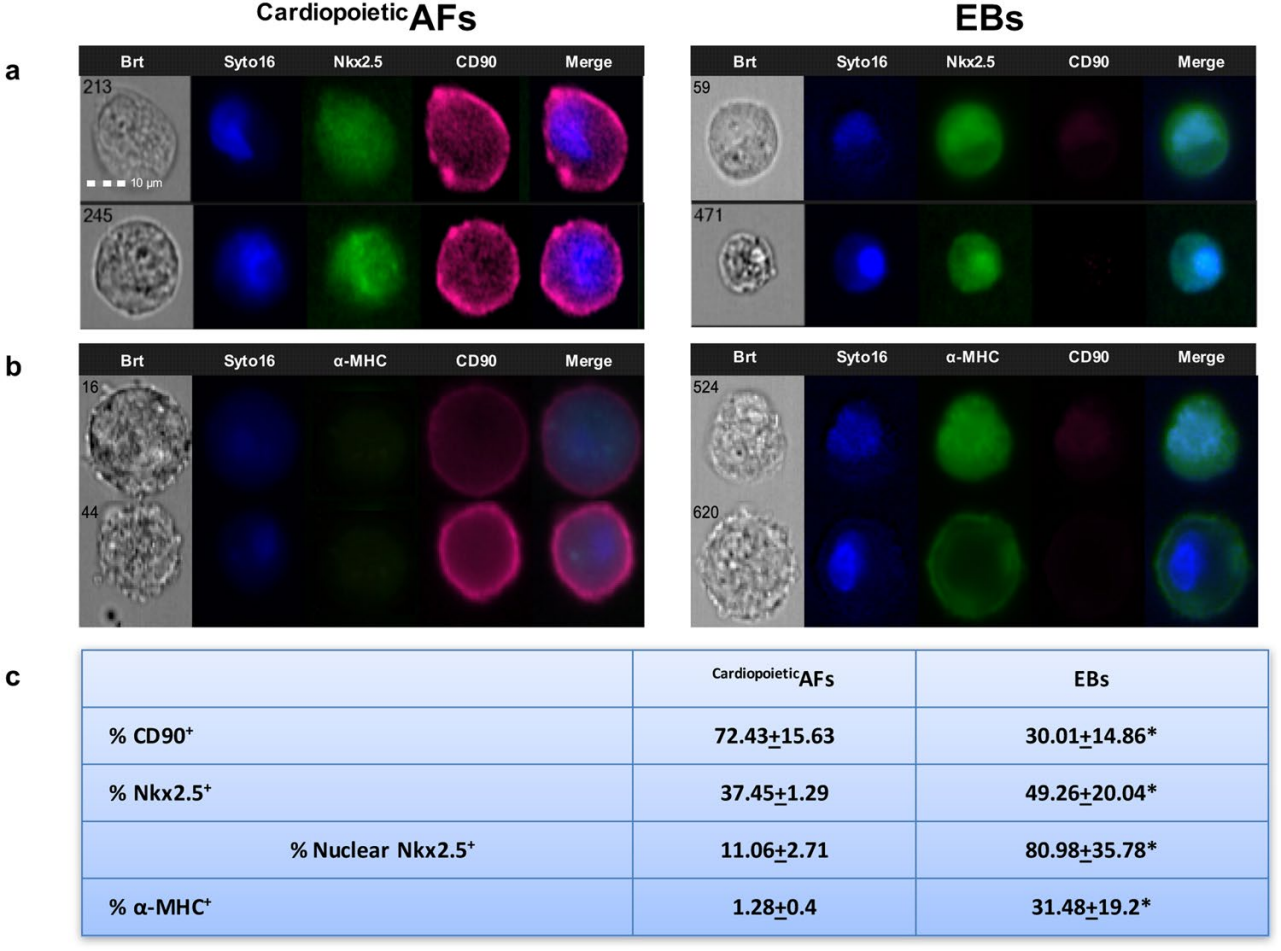
Analysis of the cardiac potential of ^Cardiopoietic^AF cell-derived EBs. Representative ImageStream images of ^Cardiopoietic^AF and ^**Cardiopoiet**ic^AF cell-derived EB cells labeled with anti-CD90 (fuchsia)/anti-Nkx2.5 (green) (**a**) and with anti-CD90 (fuchsia)/anti-α-MHC (green) (**b**). Nuclei were counterstained with Syto16 (blue). Bars: 10 µm. (**c**) % of CD90^+^, Nkx2.5^+^, nuclear Nkx2.5^+^ and α-MHC ^+^ cells are expressed as the mean ± SD. *indicates values significantly different from ^Cardiopoietic^AF. Data are representative of seven independent experiments.

It has already been described that hAF cells, such as ESCs, can give rise to EBs^16^, three-dimensional aggregates representing one of the hallmarks of pluripotent stem cells and an optimal starting point for *in vitro* lineage-specific differentiation. When we tested the different samples for their ability to form EBs, we obtained three-dimensional aggregates only from the AF samples in which cells expressed SSEA4, OCT4 and CD90 but not from the samples characterized by a low cellular expression of these markers (Table 1). We then analyzed EBs after 15 days in culture by ImageStream, an instrument that combines the phenotyping abilities of flow cytometry with the morphological details of microscopy, by producing images of each cell directly in flow. As shown in Fig. 1, this analysis showed a decrease in CD90 expression and a slight, but significant, induction of the cardiac transcription factor Nkx2.5 in hAF cell-derived EBs. Moreover, among the Nkx2.5^+^ cells, there was a dramatic increase in the nuclear localization of this transcription factor. In parallel, we analyzed the expression of α-MHC, a “late” cardiac marker; the analysis demonstrated that about one-third of the cells were α-MHC^+^. These observations suggest that only hAF cell samples expressing SSEA4, OCT4 and CD90 can give rise to EBs and that these aggregates provide a suitable microenvironment for the cardiac differentiation of some residing cells: we designated these samples as ^Cardiopoietic^AF. However, in our culture conditions, the efficacy of obtaining CM-like cells from ^Cardiopoietic^AF was very low. Moreover, using ImageStream, we observed that several cells inside the EB displayed condensed nuclei, a typical marker of apoptosis.

To overcome these limitations, we cultured hAF samples in monolayers by modifying differentiation protocols that are routinely successfully used in generating high-efficiency beating CMs from hiPS cells^23^. The hAF cells were sequentially exposed to BMP4 and Activin A for mesodermal induction, then to VEGF to drive the cells toward the cardiac lineage (myocardial induction) and finally only to ascorbic acid and 5-Aza for cardiac expansion and maturation. While these treatments induced cell damage (vacuolization, cell shrinkage, cell death, data not shown) in the samples with negative/low expression of SSEA4, OCT4 and CD90, ^Cardiopoietic^AF cells successfully underwent all the steps of the differentiation protocol. The expression of “early” and “late” cardiac-specific proteins was then analyzed by Western blot, flow cytometry and immunofluorescence microscopy.

### Induction of cardiac differentiation affects the expression and localization of the cardiac nuclear factors GATA4 and Nkx2.5 in ^Cardiopoietic^AF cells

**Figure 2:**
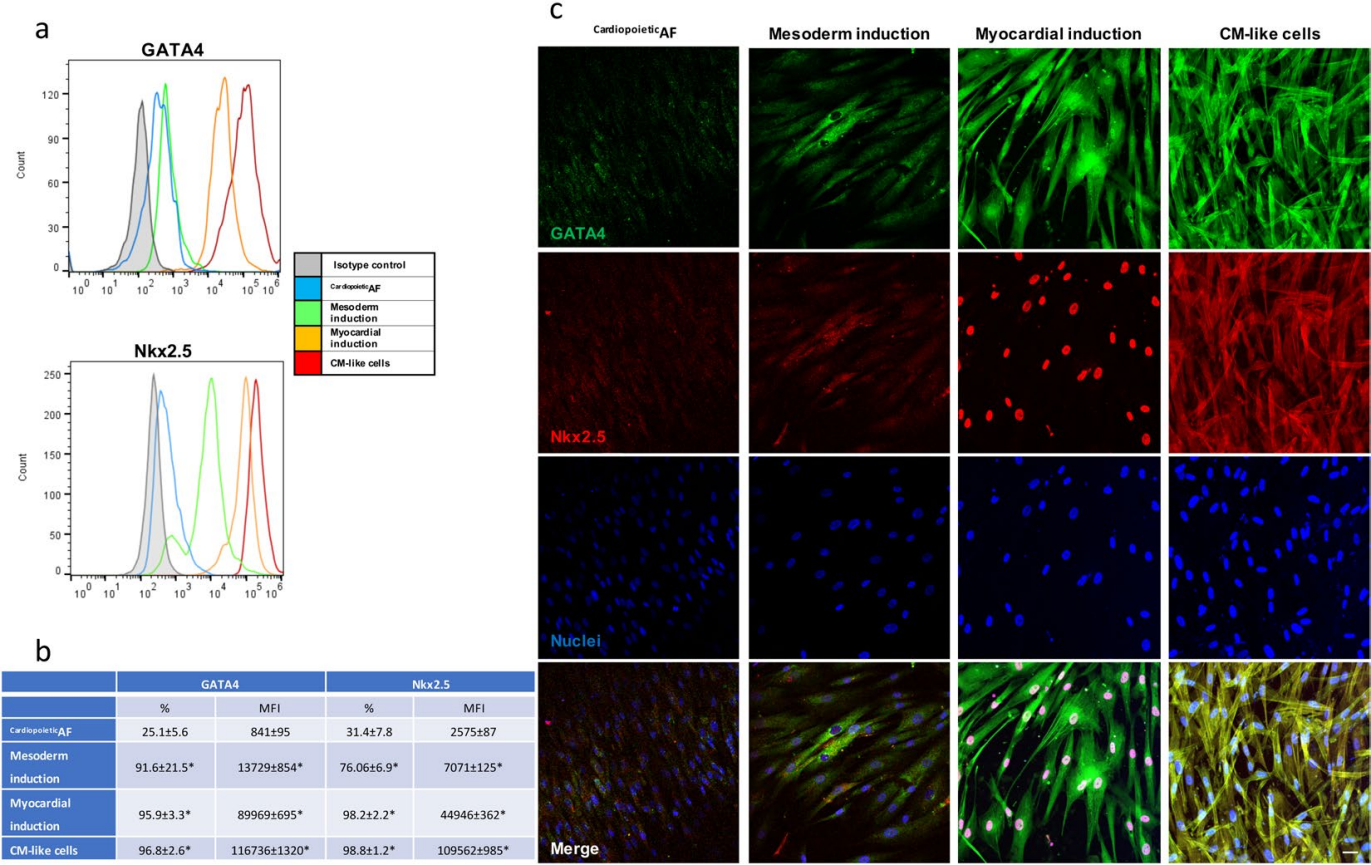
Cytometric and immunofluorescent analyses of the expression and localization of the early cardiac markers GATA4 and Nkx2.5 during the different phases of the differentiation protocol. ^Cardiopoietic^AF: before the differentiation process; mesodermal induction: after 24 hrs of activin A and BMP4; myocardial induction: after 72 hrs of VEGF; cardiomyocytes: after 15 days of differentiation. (**a**) Flow cytometry histograms: gray line, isotype control; blue line, ^Cardiopoietic^AF; green line, mesodermal induction; yellow line, myocardial induction; red line, cardiomyocytes obtained after 15 days of differentiation. (**b**) % of positive cells and mean fluorescence intensity at each differentiation step. Data are expressed as the mean ± SD. (**c**) Immunofluorescent staining for GATA4 (green), Nkx2.5 (red) and nuclei (blue) during the different phases of the differentiation protocol. The bottom line shows the merged fluorescence, as indicated. Scale bar: 10 μm. Data are representative of seven independent experiments.

The expression of the “early” cardiac markers GATA4 and Nkx2.5 was monitored during the different phases of the differentiation protocol (Fig. 2). Flow cytometry, Western blot and immunofluorescence showed that the exposure of ^Cardiopoietic^AF cells to BMP4 and activin A induced a dramatic increase in GATA4 and Nkx2.5 expression, mainly at the cytoplasmic level. After myocardial induction by VEGF, cells still overexpressed these nuclear factors, but microscopic observation documented their massive translocation into the nuclei. After 15 days of differentiation, GATA4 and Nkx2.5 were still highly expressed, even though they were mainly confined in the cytoplasm.

### Structural and functional cardiac protein expression after differentiation

**Figure 3:**
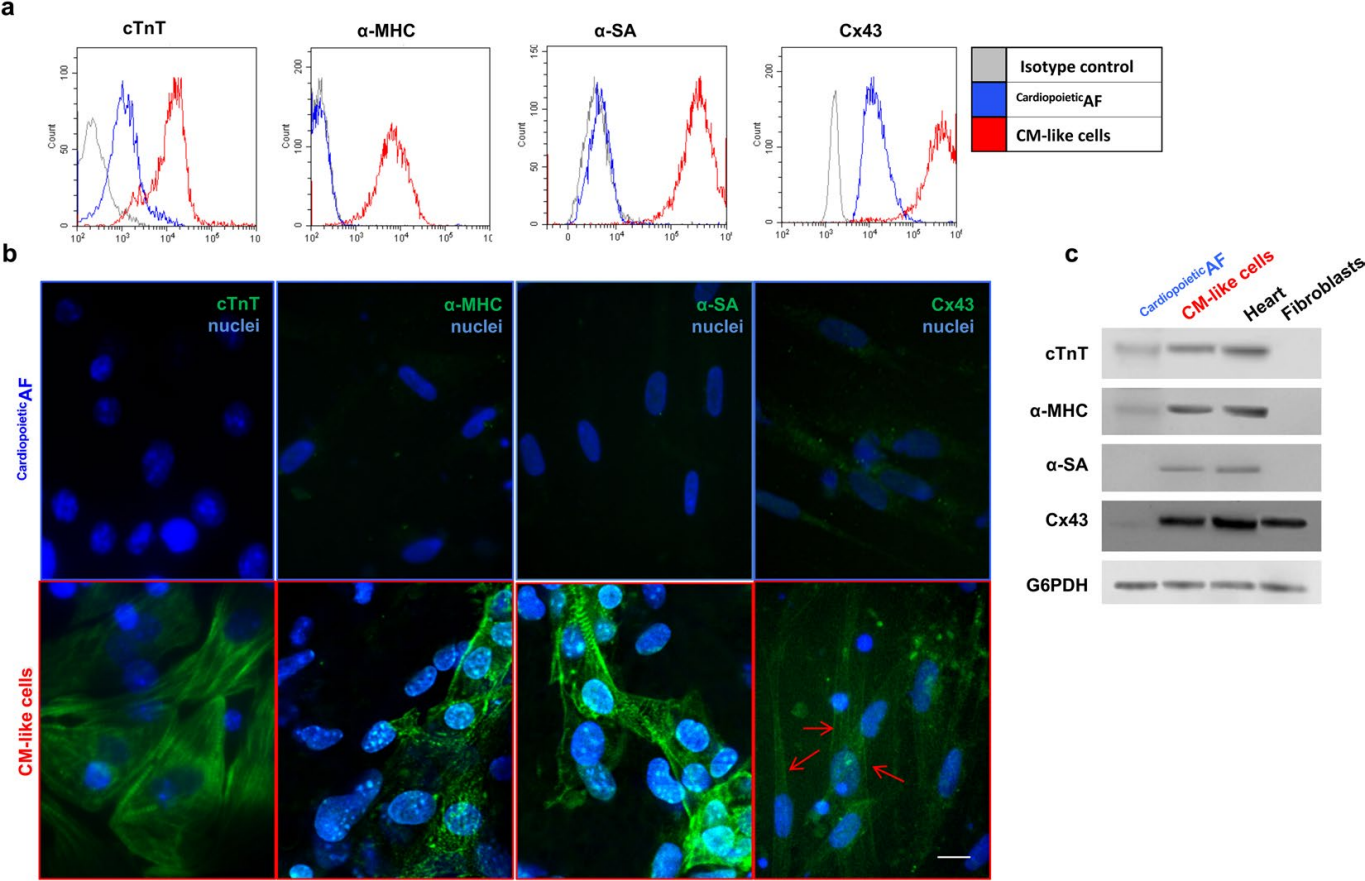
Detection of cTNT, α-MHC, α-SA and Cx43 in ^Cardiopoietic^AF cells and in CM-like cells obtained after 15 days of differentiation. (**a**) Flow cytometry analysis of TnT, α-MHC, α-SA and Cx43 in ^Cardiopoietic^AF cell (blue line) and CMs (red line). (**b**) Immunofluorescent staining for TnT, α-MHC, α-SA and Cx43 (green), as indicated. The nuclei are counterstained with DAPI (blue). Note the typical sarcomeric striations of α-SA. Red arrows indicate Cx43 localization on the cell membrane. Scale bar: 10 μm. (**c**) Western blot analysis of the cardiac markers. Human heart and fibroblast lysates were used as controls. The G6PDH lane is used as a loading control. Data are representative of seven independent experiments.

After 15 days of culture in the differentiation conditions described above, ^Cardiopoietic^AF cells changed their morphology, becoming more elongated (cell size: 39.5 ± 8.4 × 12.65 ± 3.4 μm vs 97.24 ± 34.25 × 9.6 ± 5.6 μm before and after differentiation, respectively, *p* < 0.05). We then analyzed these cells for the expression of specific “late” cardiac markers, such as sarcomeric proteins and Cx43. Interestingly, differentiated cells expressed high levels of cTnT, α-MHC and α-SA (Table 2 and Fig. 3); in some cells, α-SA displayed typical sarcomeric striations. Additionally, the expression of Cx43, which weakly marked the cytoplasm of undifferentiated ^Cardiopoietic^AF cells, dramatically increased after differentiation and moved to the cell periphery, suggesting a specific localization of this protein at the membrane level (Table 2 and Fig. 3).

**Table 2:** Cell Positivity (%) of ^Cardiopoietic^AF cells before and after differentiation.

	^Cardiopoietic^AF cells (%)	CMs (%)
α-MHC	0.9 ± 0.5	89.6 ± 8.1*
α-SA	4.5 ± 3.7	84.7 ± 9.7*
cTnT	3.1 ± 2.5	84.9 ± 10.1*
Cx43	42.8 ± 10.8	81.5 ± 10.2*
CACNA1C	15.7 ± 9.3	85.8 ± 9.8*
SERCA2	46.6 ± 12.8	84.1 ± 11.5*
RYR	1.2 ± 1.2	25.1 ± 6.6*
MYL7	0.5 ± 0.3	65.7 ± 11.3*
IRX4	0.4 ± 0.5	15.1 ± 4.4*

Data are expressed as the mean ± SD *p < 0.05 relative to ^Cardiopoietic^AF cells.

**Figure 4:**
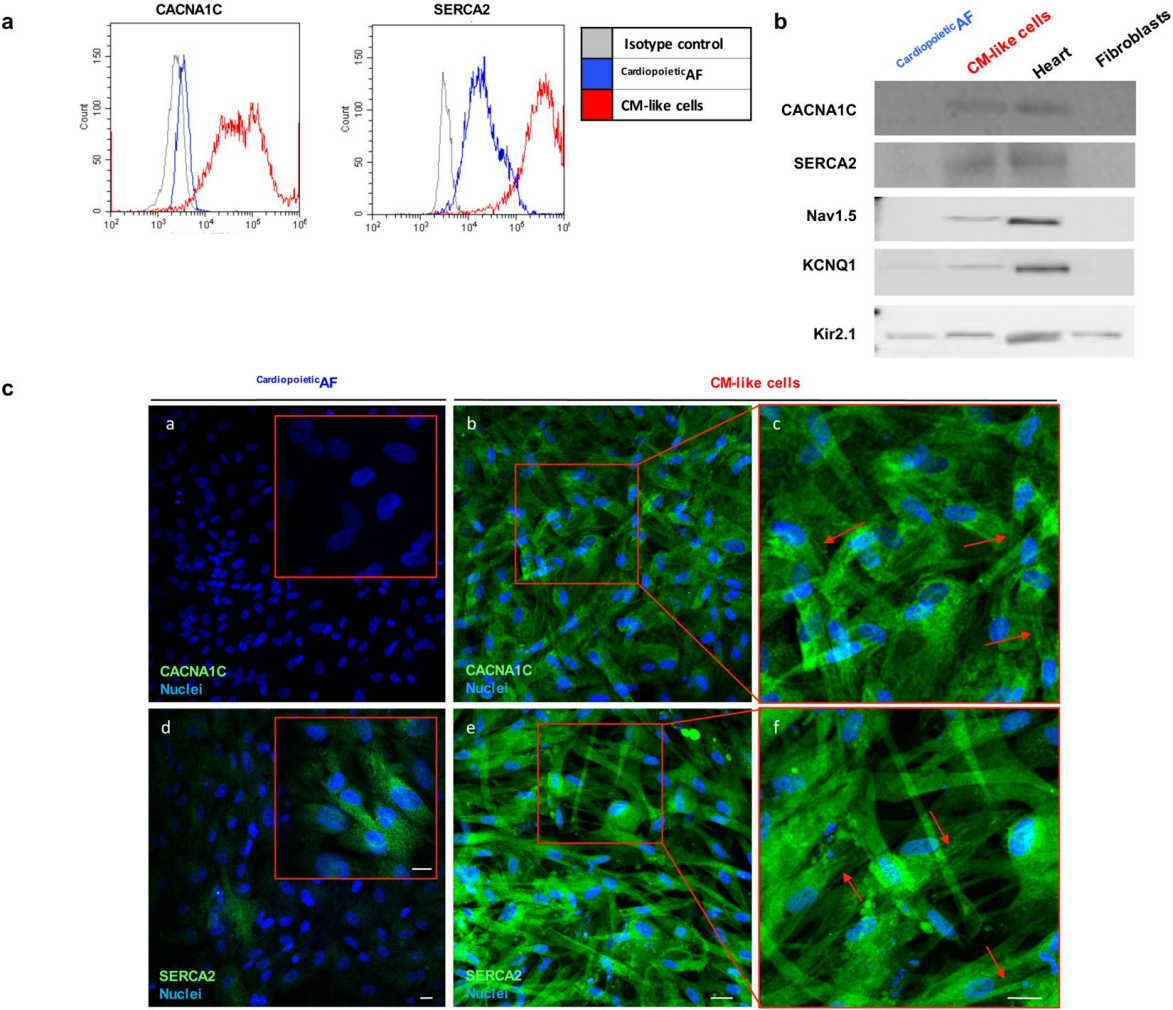
Detection of calcium pumps CACNA1C and SERCA2 in ^Cardiopoietic^AF cells and in CM-like cells obtained after 15 days of differentiation. (**a**) Flow cytometry and (**b**) Western blot analysis for CACNA1C, SERCA2, Nav1.5, KCNQ1, Kir2.1 in ^Cardiopoietic^AF cells and CMs. In Western Blot human heart and fibroblast lysates were used as controls. In the histogram of the cytometric analysis, the gray line represents the isotype control, the blue line represents ^Cardiopoietic^AF cells and the red line represents CMs. (**c**) Immunofluorescent staining for CACNA1C and SERCA2 (green) in ^Cardiopoietic^AF cells (**a** and **d**) and in CMs (**b**–**f**). Inserts in a and d represent a higher magnification; the red squares in (**b**,**e**) are magnified in c and f. Nuclei are counterstained with DAPI (blue). Red arrows indicate the cell membrane localization of CACNA1C and the sarcoplasmic localization of SERCA2. Scale bar: 10 μm. Data are representative of seven independent experiments.

We also observed by Western blot, flow cytometry and immunofluorescence the dramatic induction of the CACNA1C and SERCA2 proteins, two calcium pumps involved in excitation/contraction coupling that reside in the sarcolemma and sarcoplasmic reticulum of CMs, respectively (Table 2, Fig. 4). In differentiated cells, CACNA1C localized mainly in the perinuclear area, and in some cells (approximately 20%), it marked the cytoplasmic membrane, probably indicating a homing of the protein in the sarcolemmas of more mature CMs; SERCA2 immunolabeling changed from diffuse cytoplasmic dots in ^Cardiopoietic^AF cells into reticulated organization that resembled the sarcoplasmic reticulum (Fig. 4c). Beyond the expression of calcium pumps CANA1C and SERCA2, we evidenced by western blot that ^Cardiopoietic^AF derived CM-like cells expressed also other voltage gated ion channels that are central in the cardiac action potential: in particular, we detected the presence of the Na(+) channel Nav1.5 that is involved in the initiation and conduction of action potentials, the “delayed rectifier” potassium channel KCNQ1 which is required for repolarization phase of the cardiac action potential, and inward-rectifier potassium ion channel Kir2.1 that contributes to phase 3 repolarization and plays a major role in setting the resting membrane potential of CMs (Fig. 4b).

**Figure 5:**
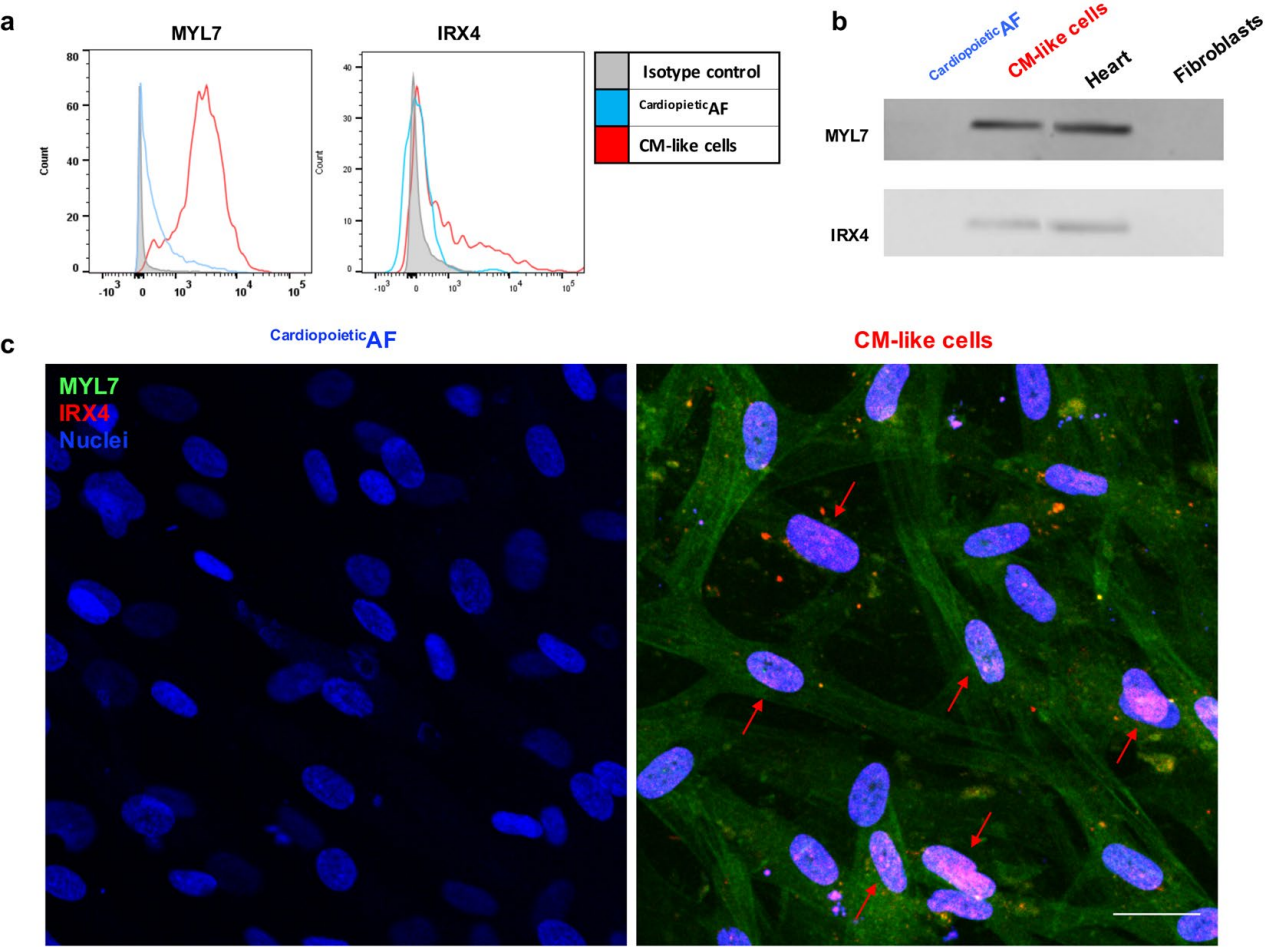
Detection of the atrial marker MYL7 and of the ventricle-specific nuclear factor IRX4 in ^Cardiopoietic^AF cells and in CM-like cells obtained after 15 days of differentiation. (**a**) Flow cytometry and (**b**) Western blot for MYL7 and IRX4 in ^Cardiopoietic^AF cells and CMs. In Western Blot human heart and fibroblast lysates were used as controls. In the histogram of the cytometric analysis, the gray line represents the isotype control, the blue line represents ^Cardiopoietic^AF cells and the red line represents CMs. (**c**) Merged immunofluorescent staining for MYL7 (green) and IRX4 (red) and nuclei (blue) in ^Cardiopoietic^AF cells (left panel) and in CMs (right panel). Red arrows indicate the nuclear localization of IRX4 in the differentiated cells. Scale bar: 10 μm. Data are representative of seven independent experiments.

Finally, to better characterize the nature of the CM-like cells obtained from ^Cardiopoietic^AF cells cultured in a monolayer, we verified the expression of atrial myosin light chain 7 (MYL7) and the ventricular-specific transcription factor iroquois homeobox 4 (IRX4). Western blot, flow cytometry and immunofluorescence showed that cardiac differentiation resulted in the upregulation of both of these proteins (Fig. 5 and Table 2); in particular, while the ventricular marker showed only a slight, albeit significant, upregulation, we observed a dramatic increase in the expression of the atrial marker MYL7. The morphological study demonstrated that the contractile protein MYL7 localized in the cytoplasm of the differentiated cells, where the signal was organized in longitudinal fibers; the transcription factor IRX4, on the other hand, was confined to the nuclei of the positive cells (Fig. 5c).

### CM-like cells generated from ^Cardiopoietic^AF cells may present spontaneous beating foci and show functional characteristics of immature CMs

**Figure 6:**
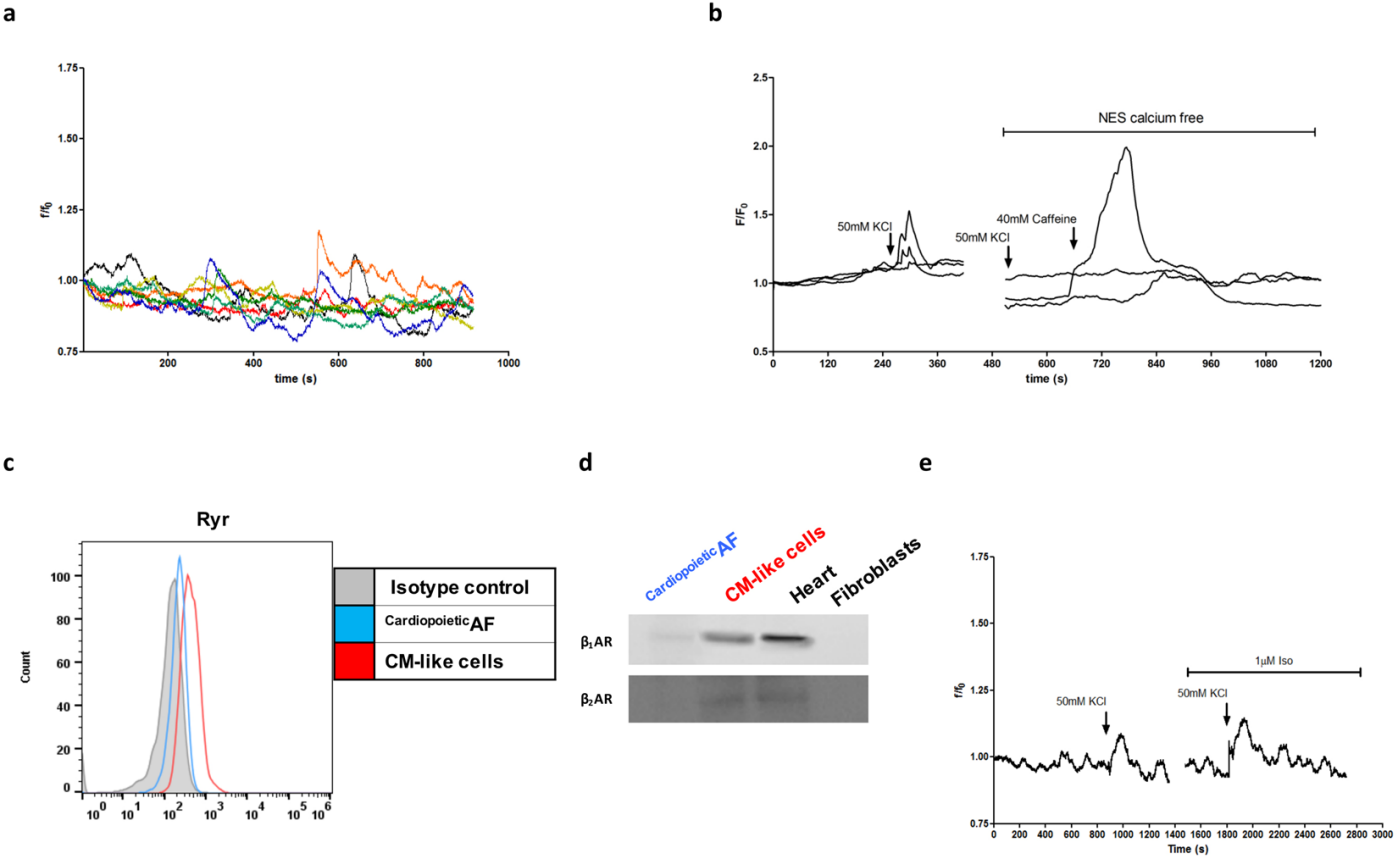
Intracellular Ca^2+^ variations and expression of RyR and β receptors in ^Cardiopoietic^AF cell-derived CM-like cells. (**a**) Spontaneous Ca^2+^ oscillations recorded in ^Cardiopoietic^AF cell-derived CMs. (**b**) CM intracellular Ca^2+^ variations in response to 50 mM KCl and 40 mM caffeine in the presence or absence of extracellular calcium (NES calcium free). (**c**) Flow cytometry of RyR expression. Gray line: isotype control; blue line: ^Cardiopoietic^AF cells; red line: CMs. (**d**) Western blot for β _1_AR and β _2_AR in ^Cardiopoietic^AF cells and CMs. Human heart and fibroblast lysates were used as controls. (**e**) Effect of 1 M isoproterenol (Iso) on 50 mM KCl-induced depolarization in CM. Data are representative of 7 different experiments.

Although after differentiation, the majority of cells expressed large amounts of sarcomeric proteins and some specific proteins involved in cardiac excitation/contraction coupling, foci of spontaneous contraction were only rarely observed (Supplementary Video S1). We then investigated the physiological properties of the CM-like cells generated from ^Cardiopoietic^AF cells. First, using fluorescence video imaging, we demonstrated that approximately 30% of the cells presented spontaneous intracellular Ca^2+^ waves (Fig. 6a, Supplementary Video S2). Then, we verified whether the cardiopoietic AF–derived CM-like cells displayed cardiac features of the excitation-contraction mechanism. Cells were stimulated with 50 mM KCl, a depolarizing agent that allows the calcium entry into the cells by the activation of the L-type Ca^2+^ channels present on the sarcolemma. In the presence of external Ca^2+^, approximately 35% of cells showed an intracellular Ca^2+^ increase; this response was not observed when, as a control, cells were stimulated in the absence of extracellular Ca^2+^. We then checked for the presence and activity of the Ryanodine receptors (RyRs), channels essential to cardiac contraction because they mediate the outflow of calcium ions from the sarcoplasmic reticulum. Flow cytometry analysis of CM-like cells generated from ^Cardiopoietic^AF cells showed that 25% of cells expressed RyRs (Fig. 6c). To test the maturity of these calcium channels, cells were stimulated with 40 mM caffeine, an RyR agonist. After treatment, approximately 20% of cells bathed in Ca^2+^-free medium responded by increasing intracellular Ca^2+^ (Fig. 6b). These data suggested that when expressed, RyRs were also active and responsive to specific agonists.

We next sought to determine whether β-adrenergic signaling, a canonical CM signaling pathway, was operational in ^Cardiopoietic^AF cell-derived CM-like cells. Western blot analysis demonstrated that differentiation was accompanied by increased expression of β1-AR, while β2-AR was weakly or not modulated (Fig. 6d). When we tested the responsiveness of the cells to isoproterenol, a nonselective β-adrenergic receptor agonist that enhances Ca^2+^ influx by phosphorylating L-type Ca^2+^ channels, we observed a weak potentiation in the KCl-evoked intracellular Ca^2+^ increase in approximately 10% of cells (Fig. 6e). These results suggest that β-adrenergic receptors and their associated intracellular signaling partners are present and functional only in a portion of CM-like cells derived from ^Cardiopoietic^AF cell samples.

## Discussion

The ideal goal of stem cell therapy is to substitute necrotic or dysfunctional cardiac tissue with new competent CMs derived from stem cells. To date, the only source that has given unambiguous results about cardiogenic potential are ES^23–25^ and iPS cells^11,23,26,27^, but ethical barriers and clinical concerns have hampered their use in the medical context^28^. hAF cells could circumvent these limits and provide effective cell replacement therapy for cardiac diseases, particularly for application to congenital heart defect repair^14,18^. Here, we characterized a population named ^Cardiopoietic^AF cells, with a profile of pluripotency that may give rise to a cell population that displays morphological and functional features of CMs.

We previously demonstrated that unselected second trimester AFs may form EBs expressing stem cells specific genes characteristic of ESC^16^. In this study, we confirmed and extended this observation, demonstrating that ^Cardiopoietic^AF cell samples, such as ESCs, can give rise to EBs with cardiogenic potential, as evidenced by the significant increase in both Nkx2.5 expression and nuclear translocation. However, this method, which is generally used for the cardiac differentiation of ESCs and iPS cells, does not seem to be as efficient in generating CMs from AF cells because only approximately 30% of the differentiated progeny expressed the “late” cardiac marker α-MHC. This finding is unsurprising because, although EBs are widely considered an optimal starting point for *in vitro* lineage-specific differentiation, the EB-mediated differentiation protocol has some important limitations^29^. The efficiency, indeed, is dependent on EB size because larger EBs tend to differentiate toward mesoderm and endoderm, while smaller EBs direct their differentiation toward ectoderm: the lack of uniformity in EB size, resulting in nonhomogeneous and asynchronous differentiation of the residing cells^30^ hampers their effective employment in regenerative medicine. Moreover, we observed that in our model, the EB cells often presented condensed nuclei. This hallmark of apoptosis is probably due to the fact that cells within the aggregates suffer from limitations in the diffusion of nutrients and gas in the static culture environment. Laflamme *et al*.^25^ have already shown that cardiac differentiation from ES cells was more efficient in suspension and monolayer cultures than in EB-based methods. Because ^Cardiopoietic^AF cells exhibit some aspects of ES cells, we tried to drive the ^Cardiopoietic^AF cells toward the cardiac lineage using a directed differentiation approach in monolayers.

Most of the experiments on the differentiation potential of hAF cells reported in the literature have been carried out by isolating CD117^+^ cells from the initial heterogeneous population. Data on the surface markers of AF cells are not consistent among reports from different groups, and the results for the expression of c-kit/CD117 in AF cells are mixed: some research demonstrated positive gene expression by PCR, but flow cytometry results were negative or only slightly positive^21,31–33^. Moreover, considering that when detected, CD117^+^ cells represent only approximately 1–5% of AF cells, whereas the percentage of cells expressing pluripotent markers such as Oct4 or SSEA4 can be sensibly higher, the preferential selection of the c-kit^+^ subpopulation might be too restrictive to determine the possible loss of cells at the top of the stem hierarchy with high differentiation potential. Thus, it is still unclear whether sorting for CD117 expression is necessary or appropriate. A recent study reported that the unsorted population was more efficiently differentiated toward a neuronal lineage, whereas the c-kit-sorted population was better specified toward adipogenic, osteogenic, and chondrogenic lineages^34^. These findings indicate that further research is needed to determine the appropriate isolation and culture procedures for the diverse differentiative endpoints. In any case, because the cells contained in AF are a heterogeneous mixture of many cell types, the analysis of undifferentiated AF cell samples is necessary to characterize the starting populations in differentiation experiments. Our data indicate that a subset of AF cell samples expressing pluripotent and multipotent stem markers - the ^Cardiopoietic^AF cell samples - can differentiate into CM-like cells that, as discussed below, recapitulate some functional aspects of iPS- and ESC-derived CMs. This possibility to develop *in vitro* cardiogenic potential without resorting to any immunoselective method is pivotal: the absence of the use of xenogenic antibodies is indeed crucial in advancing cell therapy applications^35^.

As reviewed by Petsche Connel^22^, other groups have also reported that AF cells could be guided toward cardiac differentiation: rat or human c-kit^+^-selected cells may acquire a CM phenotype when co-cultured with rat neonatal CMs, and functional communications develop among the cells of the co-culture^14,17^. However, this differentiation process occurs with a very low yield and presents limits deriving from a xenogenic co-culture sysytem^36^. Other evidence suggests that the modulation of WNT^15^ signaling or exposure to 5-Aza^14,15^ may induce a CM phenotype in unselected AF cells, but these protocols were also characterized by low differentiation efficiency, and organized sarcomeric structures and spontaneous contraction were not detected. This partial differentiation is probably because modulation of WNT signaling or the application of demethylating agents are not able, alone, to fully commit hAF cells to a cardiac lineage^14,15,19^. Yang and colleagues^37^ demonstrated that stage-specific addition of morphogenic and growth factors such as activin A, BMP4, FGF and VEGF led to the gradual transition of ES cells to mesoderm, eventually to form cardiovascular progenitors. Adapting this protocol, we obtained cells expressing several cardiac-specific markers from unselected ^Cardiopoietic^AF.

During the first phases of differentiation, we detected the upregulation and nuclear translocation of the cardiac nuclear factors GATA4 and Nkx2.5, which probably triggered the synthesis of “late” cardiac markers, such as specific structural and functional proteins. Moreover, ^Cardiopoietic^AF cell-derived CM-like cells changed their aspect, developing an oblong/cylindrical morphology that recalled the shape and size of mature CMs. Our protocol results in the production of both atrial and ventricular myocytes, as testified by the expression of MYL7 and IRX4, two well-known cardiac-specific differentiation markers^38^.

To contribute to cardiac function, newly generated CMs should be not only fully differentiated but also electrically integrated within the surrounding native cardiac tissue. Following the differentiation protocol, we observed upregulation of Cx43, localized at the cytoplasmic and membrane levels. Cx43 is a dominant connexin expressed in cardiovascular tissue and is a key component of gap junctions. One of the shortcomings of current patch materials for congenital heart defect repair is that they are non-conductive^39^, which leads to an increased risk of arrhythmias and sudden cardiac death. The observation that upon differentiation, Cx43 also localized in the cell membrane indicates the possible initial formation of gap junctions. Although an electrical connection with neighboring cardiac cells will not be sufficient to avoid cardiac arrhythmias if the cells are not able to propagate the action potential at the same speed as the surrounding cells, this finding is a pivotal step in the maturation process.

Voltage gate ion channels are critical for all aspects of cardiac function, including rhythmicity and contractility. In the heart, prominent voltage-gated ion channels include sodium, potassium and calcium channels. We found that ^Cardiopoietic^AF derived CM-like cells also slightly expressed Nav1.5 that is involved in the initiation and conduction of action potentials, the “delayed rectifier” KCNQ1 which is required for repolarization phase of the cardiac action potential, and the inward-rectifier potassium ion channel Kir2.1 that contributes to repolarization and to the setting of the resting membrane potential.

CMs are characterized by transient increases in intracellular Ca^2+^ concentration, which are responsible for activating contraction. When an action potential depolarizes the cell membrane, voltage-gated Ca2^+^ channels (L-type calcium channels) are activated, and the resulting Ca2^+^ influx activates RyRs on the sarcoplasmic reticulum membrane, which causes more Ca2 + to be released into the cytosol. This process, also called Ca^2+^-induced Ca^2+^ release, involves numerous ion channels, transporters and regulatory enzymes. In ^Cardiopoietic^AF cell-derived CM-like cells, we found an increase in L-type calcium channel (CACNA1) expression. These channels appeared localized in both the cytoplasmic compartment and at the membrane level. Additionally, SERCA2 increased its expression in differentiated cells, depicting reticular structures that resembled the sarcoplasmic reticulum. Moreover, we found that approximately one-quarter of ^Cardiopoietic^AF cell-derived CM-like cells expressed RyRs. These data are in accordance with our functional analyses, in which the presence of spontaneous calcium oscillations and responsiveness to a depolarizing agent were evident, although at different amplitudes of calcium transients. We also demonstrated that 20% of cells were caffeine responsive, namely, cells in which RyRs are capable of loading and unloading Ca^2+^. This is indicative of the fact that we obtained CM-like cells with different levels of maturity, some of which displayed features characteristic of cardiac excitation-contraction with efficient Ca^2+^-induced Ca^2+^ release. The finding of different levels of maturity is unsurprising: also in hES cell-derived CMs, only few cells are caffeine responsive and a great variability in RyR expression is observed, moreover representing only a small fraction (0.1%) of the adult level^40,41^. In addition, when compared to adult CMs, hES cell-derived CMs exhibited smaller whole-cell peak amplitudes and slower upstroke and decay in calcium transients in response to caffeine^42^. Finally, data from hiPS cell-derived CMs indicate the presence of spontaneous calcium transients associated with immature calcium-handling properties^11,43^.

Isoproterenol, a *β*-adrenergic receptor agonist, is known to activate many intracellular targets involved in the control of excitation–contraction coupling and Ca^2+^-induced Ca^2+^ release. Evidence that ^Cardiopoietic^AF cell-derived CM-like cells are functionally immature was also revealed by results from intracellular calcium measurements in the presence of isoproterenol. Indeed, we observed that in approximately 35% of the cells, L-type Ca^2+^ channels responded to a depolarizing agent such as KCl, one-third of which (approximately 10% of the total population) were also responsive to isoproterenol. This observation is in accordance with previous results showing the same immature functional phenotype in hES and hiPS cells, in which the inotropic response appeared to be not affected by isoproterenol^11,44^.

Recently, Jiang *et al*.^45^ showed that hAFs can be reprogrammed and subsequently used to generate functional CMs with robust and highly organized myofilament structures. In this study, we demonstrated that if the hAFs present the cardiopoietic phenotype (high expression of OCT4, SSEA4, CD90), it is possible to obtain CM-like cells that recapitulate the iPS- or ES-cell-derived CM phenotype with a time- and cost-saving method that avoids cell reprogramming and all the risks related to iPS cell utilization^11^.

In conclusion, the data presented herein suggest that unselected ^Cardiopoietic^AF might be induced to differentiate into cells that express several cardiac-specific proteins and recapitulate some electrophysiological features of ES or iPS-cell-derived CMs, showing beating foci, albeit rare. Remaining challenges include the promotion of the maturation of these cells by improving their functional organization and electric properties. Addressing these challenges may require novel culture methods that better resemble the *in vivo* niche, and more attention must be paid to paracrine signaling and the cellular milieu. Despite these limitations, ^Cardiopoietic^AF cells demonstrate potential as a tool to enhance basic biological understanding, to improve *in vitro* drug screening, and thus, to create new therapeutic options.

## Methods

AF samples were obtained from 15 women undergoing amniocentesis for prenatal diagnosis at 16–17 weeks of pregnancy after written informed consent, in accordance with the Declaration of Helsinki. All samples had normal diploid male karyotypes, as evidenced by cytogenetic investigation. The mean (±SD) maternal age at amniocentesis was 37.9 ± 2.6 years (Table 1 and Supplemental Fig. 1). The study was approved by the ethics committee of the “G. d’Annunzio” University of Chieti-Pescara, ASL Lanciano-Chieti-Vasto, Italy and all experiments were performed in accordance with relevant guidelines and regulations.

### Isolation and culture of hAF cells

hAF cells, isolated as previously described^16^, were cultured in Iscove’s modified Dulbecco’s medium (IMDM, Lonza Group Ltd, Basel, CH) supplemented with 20% fetal bovine serum (FBS, GE Healthcare HyClone Defined Fetal Bovine Serum, Buckinghamshire, UK), 100 U/ml penicillin, 100 µg/ml streptomycin, 2 mM L-glutamine, (all Sigma-Aldrich, Saint Louis, USA) and 5 ng/ml basic fibroblast growth factor (bFGF, Invitrogen, Thermo Fisher Scientific, Waltham, MA, USA) and incubated at 37 °C with 5% humidified CO_2_.

### EB generation

EBs were generated with the hanging drop method, as previously described^16^. Briefly, hAF cells (6°–8° passage) were harvested, and 25 µL drops of cellular suspension (1–10 × 10^4^ cells/mL) were cultured in IMDM supplemented with 15% FBS, 100 U/ml penicillin, 100 µg/ml streptomycin, 2 mM L-glutamine, 0.1 mM 2-mercaptoethanol, 10 μM 5-aza-2′-deoxycytidine (5-Aza) and 50 μg/mL ascorbic acid (all Sigma-Aldrich) in Falcon 100 mm × 15 mm Not TC-Treated Bacteriological Petri Dish (Corning, Flintshire, UK). After 6 days in suspension, the aggregates were transferred onto gelatin-coated tissue culture plates and grown for 10 days in the same medium.

### Culture in monolayer and cardiac differentiation

hAFs were seeded onto Matrigel (Corning, Flintshire, UK)-coated plates and cultured as described above. When 80–90% confluence was reached, the medium was changed to RPMI 1640 (Life Technologies) supplemented with B-27 minus insulin (GIBCO, Thermo Fisher Scientific), 50 μg/mL ascorbic acid, 10/M 5-Aza, and differentiation was induced by exposure for 24 hr to activin A (50 ng/mL, R&D Systems) and BMP4 (25 ng/mL, R&D Systems), followed by a 72 hr treatment with VEGF (10 ng/mL, R&D Systems). Cells were analyzed after 15 days of differentiation.

### Flow cytometry and imaging flow cytometry

For flow cytometry and imaging flow cytometry, cells were treated with the FIX & PERM® Kit (Thermo Fisher Scientific) and then incubated for 1 hr at RT with anti-Gata4 (1:50; Abcam), anti-Nkx2.5 (1:50; R&D Systems), anti-α-myosin heavy chain (α-MHC; 1:100; Abcam), anti-CD90 (1:50; BD), anti-cardiac Troponin T (cTnT, 1:100; Abcam), anti-α-sarcomeric actin (α-SA; 1:100; Abcam), anti-connexin 43 (Cx43; 1:100; Santa Cruz Biotechnology), anti-Sarcolemmal Ca^2+^ channel (CACNA1C, 1:100; Abcam), anti-Sarco-endoplasmic Reticulum Ca^2+^ ATPase protein (Serca2, 1:100; Invitrogen, Thermo Fisher Scientific), anti-Myl7 (1:100; Invitrogen), anti-IRX4 (1:100; Santa Cruz Biotechnology) or anti-Ryanodine receptor (RyR, 1:200 ThermoScientific, Rockford, Illinois, U.S.A), followed by incubation with the appropriate secondary antibody conjugated with Alexa Fluor 488, PE-Alexa Fluor 647 or PE-Cy7 (1:200; Invitrogen, Thermo Fisher Scientific), as previously reported^46^. Cells incubated with isotypes (all from BD) were used as negative controls.

For imaging flow cytometry, samples were examined by an ImageStream IS100 (Amnis, Merck-Millipore) multispectral imaging flow cytometer using a 488 nm solid state laser (50 mW) and Inspire software (v 4.1.434.0). Nuclei were counterstained with Syto16. Data were analyzed using IDEAS software v 5.0 (Amnis, Merck-Millipore).

Cytometric analysis was performed with a Cytoflex cytometer (Beckman Coulter), and data were analyzed with FlowJo (TreeStar, Ashland, OR) or CytExpert Acquisition and Analysis Software.

### Immunoblot analysis

For the immunoblot analysis, cells were lysed in RIPA homogenization buffer, electrophoresed in SDS–PAGE gradient 4–12% and transferred onto membranes that were then hybridized with anti- cTnT (1:500), anti-α-MHC (1:500), anti-α-SA (1:200), anti-Cx43 (1:200), anti-CACNA1C (1:1000), anti-SERCA2 (1:1000), anti-MYL7 (1:500), anti-IRX4 (1:100), anti-β_1_ or anti-β_2_ adrenoceptor (β_1_ or β_2_AR, 1:100, Santa Cruz Technologies), anti-Nav1.5 (1:1000, Abcam), anti-KCNQ1 (1:500 ThermoFisher), anti-Kir2.1 (1:200 R&D System) or anti-G6PDH antibodies (1:1000; Santa Cruz Technologies), followed by the appropriate HRP-conjugated secondary antibodies (1:1000; Santa Cruz Technologies), as previously reported^47^. Protein expression was detected with an enhanced chemiluminescence kit (Amersham, Little Chalfont, UK) and quantified by densitometry with ImageJ Software (Supplemental Fig. S2). Human heart extract and foreskin fibroblast cell lysates (Santa Cruz Technologies) were used as positive and negative controls, respectively.

### Immunofluorescence staining and microscopy

Cells fixed with 4% paraformaldehyde and permeabilized with 0.5% Triton X-100 were incubated with anti-GATA4 (1:50), anti-Nkx2.5 (1:50), anti-cTnT (1:100), anti-α-MHC (1:100), anti-α-SA (1:100), anti-Cx43 (1:100), anti-CACNA1C (1:100), anti-SERCA2 (1:100), anti-MYL7 (1:100), or anti-IRX4 (1:100), followed by the appropriate secondary antibody conjugated with Alexa Fluor 488 or PE (1:200), as previously reported^5^. Nuclei were counterstained with DAPI. Samples treated only with the secondary antibody were used as controls. Images were acquired with an Axio Vert A1 microscope or a Zeiss LSM 7 MP multiphoton microscope (Carl Zeiss, Jena, Germany) equipped with an Axio-Examiner.Z1 upright microscope. The two-photon excitation was obtained using a Ti:Sapphire Laser Coheren Camaleon Vision II in locked mode (Coherent Inc. Santa Clara, CA). Excitations were fixed at 730 nm or 980 nm and 1087 nm for DAPI, FITC and PE, respectively. Fluorescence emission was collected using an NDD detector set in transmission mode using an SP 485 nm filter (DAPI staining) or with a BiG detector in reflection mode, equipped with BP 500–550/575–620 nm for FITC and PE. Images were analyzed by ZEN 2009 (Carl Zeiss).

### Calcium Imaging

Intracellular Ca^2+^ levels were monitored using the dye Fluo4-acetoxymethyl ester (Fluo4/AM, Life Technologies). An upright microscope (Zeiss Axio Examiner; Carl Zeiss) was used, equipped with 40 × 0.75 NA water-immersion objectives connected by optical fiber to a 75 W Xenon lamp and a monochromator (OptoScan; Cairn Instrument, UK). Sub-millisecond bandpass and wavelength controls were used with a back-illuminated camera (EMCCD, Evolve 512; Photometrics, Tucson, USA). The cells were incubated with 5 µM Fluo-4/AM in normal external solution (NES: 140 mM NaCl, 2.8 mM KCl, 2 mM CaCl_2_, 2 mM MgCl_2_, 10 mM glucose, 10 mM Hepes, pH 7.3) supplemented with 1% (w/v) bovine serum albumin for 40 min at 37 °C. Recordings on Fluo4-loaded cells were performed in normal external solution (or where indicated in NES w/o Ca^2+^ in which 2 mM Ca^2+^ was substituted with 2 mM Mg^2+^). The fluorescence was acquired by setting excitation at 488 nm and images acquired at 5 frames/s with an EMCCD camera and stored on an interfaced computer for off-line analysis using Metafluor (Molecular Device, Sunnyvale, CA, USA). The temporal analysis was calculated as the mean fluorescence intensity signal in a selected cell area, as f/f0, where f is the fluorescence emission of a single loaded cell acquired during a time lapse, and f0 is the mean fluorescence intensity of the same cell calculated from images acquired during the first 5 s.

### Statistical analysis

All quantitative data are presented as the mean ± SD. Statistical comparison was performed using one-way analysis of variance (ANOVA) and Student’s t-test. The level of significance was set at P < 0.05.

### Statement

All data generated or analyzed during this study are included in this published article (and its Supplementary Information files).

## Electronic supplementary material

Supplementary Table S1 and Figures S1 and S2

Supplementary video S1

Supplementary video S2
