# Supplementary material for: Cardiomyocytes Derived from Human CardiopoieticAmniotic Fluids
Source: Sci Rep. 2018 Aug 13;8:12028. doi: 10.1038/s41598-018-30537-z (PMC6089907; doi:10.1038/s41598-018-30537-z)

Cardiomyocytes Derived from Human Cardiopoietic Amniotic Fluids

Angela Di Baldassarre[1], Maria A D'Amico[1], Pascal Izzicupo[1], Giulia Gaggi[1], Simone Guarnieri[2], Maria A Mariggiò[2], Ivana Antonucci[3], Barbara Corneo[4], Dario Sirabella[4], Liborio Suppia[3] and Barbara Ghinassi[1*]
[1] Department of Medicine and Aging Sciences, University "G. d'Annunzio" of Chieti-Pescara, Via dei Vestini 31, 66100, Chieti, Italy
[2] Department of Neuroscience, Imaging e Clinical Scienes, University "G. d'Annunzio" of Chieti-Pescara, Via dei Vestini 31, 66100, Chieti, Italy
[3] Department of Department of Psychological, Humanities and Territorial Sciences, University "G. d'Annunzio" of Chieti-Pescara, Via dei Vestini 31, 66100, Chieti, Italy
[4] Stem Cell Core Facility, Columbia University Medical Center, 650 W. 168th St., 10032 New York, NY, USA

Supplementary Table S1. Phenotypic characterization of hAF cells samples

AF Sample	CD29	CD73	CD44	CD34	CD45	CD31	Tra1-60	SSEA4	OCT4	CD90
#1	++	++	++	-	-	-	-/+	++	++	++
#2	++	++	++	-	-	-	-	-/+	-/+	++
#3	++	++	++	-	-	-	-	-/+	-/+	-/+
#4	++	++	++	-	-	-	-	++	+	++
#5	++	++	++	-	-	-	-	++	++	+
#6	++	++	++	-	-	-	-	-	-/+	-
#7	++	++	++	-	-	-	-	-/+	-/+	+
#8	++	++	++	-	-	-	-	-/+	-/+	+
#9	++	++	++	-	-	-	-	++	+	++
#10	++	++	++	-	-	-	-	++	++	+
#11	++	++	++	-	-	-	-	+	-	-/+
#12	++	++	++	-	-	-	-	-/+	-/+	++
#13	++	++	++	-	-	-	-	-/+	-/+	-/+
#14	++	++	++	-	-	-	-	++	+	++
#15	++	++	++	-	-	-	-/+	++	++	++

Legend: - (<1%) not expressed; -/+ (1-10%) slightly expressed; +(10-50%) expressed; ++ (>50%) strongly expressed

Supplemental Figure S1: Normal diploid male Caryotype (46, XY). The figure is rappresentative of all 15 samples.

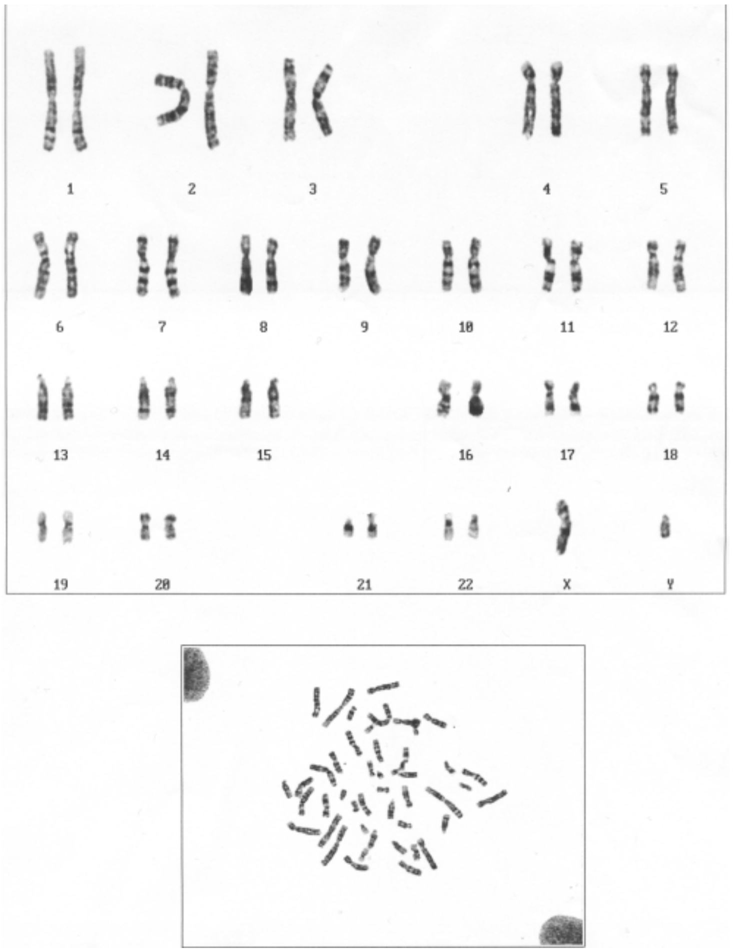

Supplemental Figure S2: Densitometry of the Western Blot analyses. Data are expressed as the mean±SD of the optical density percentage relative to the positive control (heart lysate). * indicates value of CM-like cells statistically different ($p<0.05$) from ^{Cardiopoietic}AF cells.

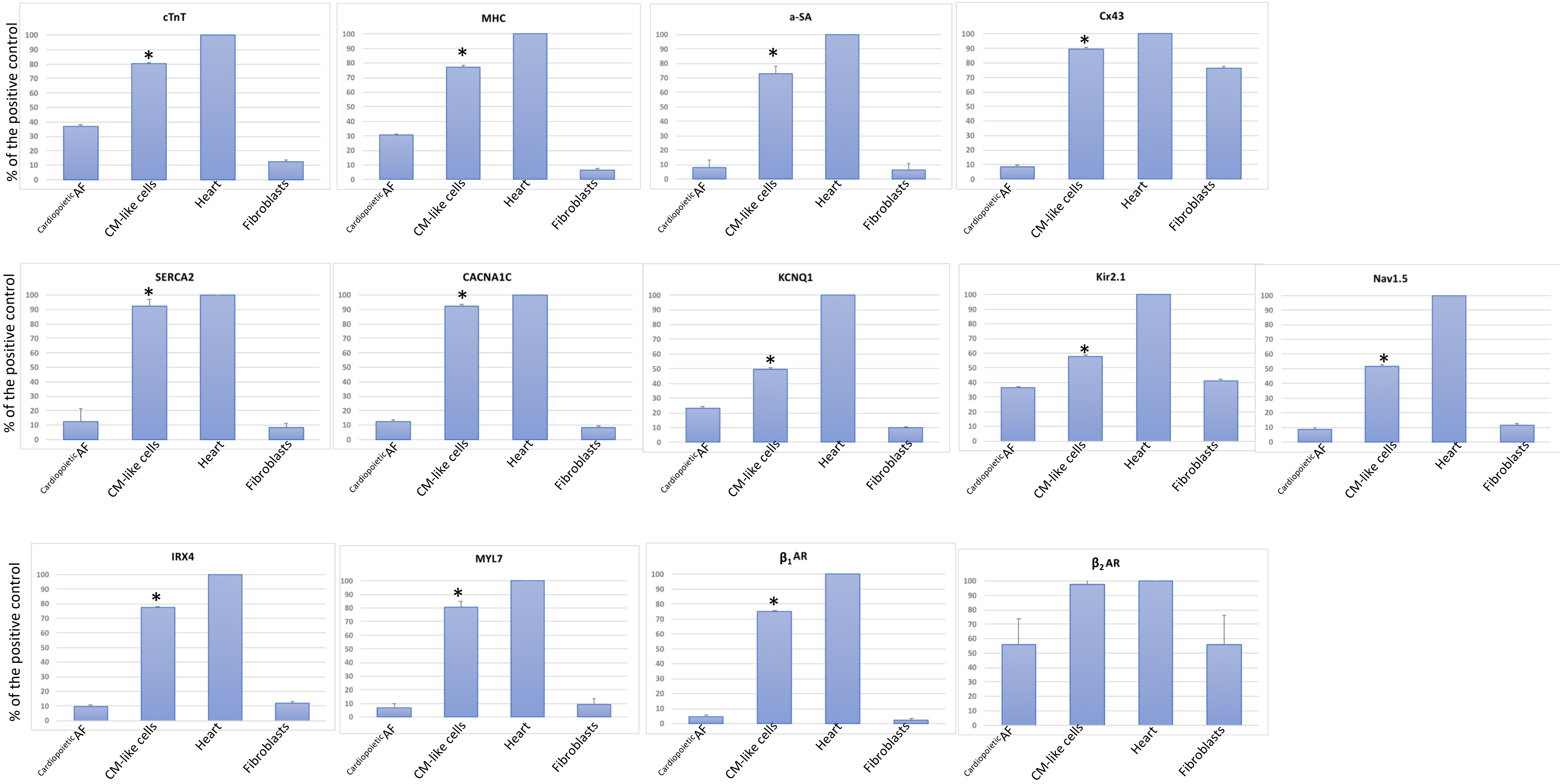

Supplement: Supplementary file 1 — Supplementary Table S1 and Figures S1 and S2 [file 41598_2018_30537_MOESM1_ESM.pdf]
